# Posaconazole for lobomycosis

**DOI:** 10.1016/j.bjid.2021.101576

**Published:** 2021-04-12

**Authors:** Alessandro C. Pasqualotto, Sergio D. Jaskulski Filho, Maria G. de Sena, Auri F. dos Santos, Marilia M.S. Severo

**Affiliations:** a Santa Casa de Misericordia de Porto Alegre, Porto Alegre, RS, Brazil; b Universidade Federal de Ciencias da Saude de Porto Alegre, Porto Alegre, RS Brazil; c Centro de Referencia em Doenças Tropicais, Macapa, PA Brazil

**Fig. 1:**
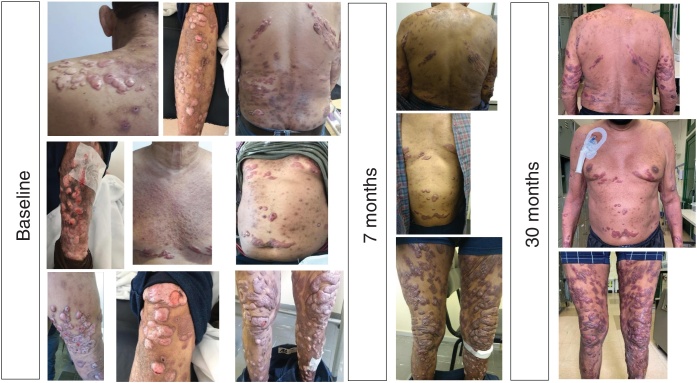
Evolution of lobomycosis lesions in a patient chronic renal failure on treatment with posaconazole.

A 57 year-old man was referred for renal transplantation due to diabetic nephropathy. He was transferred from the city of Macapá, in the Brazilian Amazonian region. He reported a history of multiple chronic keloid-like lesions since he was three years-old. Along his life these lesions progressed to involve his face, trunk, and members. Lesions varied in size, some were confluent, nodular and ulcerated. At the age of 16 he was diagnosed with lobomycosis (lacaziosis or keloidal blastomycosis), after a skin biopsy was performed. He received courses of dapsone, itraconazole and clofazimine with no success. His social life was very much impacted by the disease. At arrival in our medical center, we had extensive and itchy, disseminated keloids (Fig. 1). He was on treatment with hemodialysis but otherwise healthy. He was put on posaconazole oral solution 400 mg bid in August 2018, which was well tolerated. His skin lesions markedly improved over time, as documented 7 and 30 months after initiation of antifungal therapy. The patient himself felt much better, and reported that most lesions decreased in size, and some healed. No new lesions showed up during follow-up time. Patient is currently in list for a kidney transplantation, and posaconazole should be maintained after organ transplantation, until all skin lesions are healed. Lobomycosis is a neglected fungal disease that affects hundreds of people in the Amazon area. Therapy of lobomycosis is usually frustrating, due to side effects and lack of efficacy of available drugs. Posaconazole has demonstrated efficacy in lobomycosis^1^ even though clinical experience remains very limited. This is the first report in the literature of treatment of extensive lobomycosis with posaconazole, in a patient with chronic renal failure on dialysis. The drug promoted partial clinical response and long term-therapy is expected to result in additional benefit.
